# Direct isotopic evidence of biogenic methane production and efflux from beneath a temperate glacier

**DOI:** 10.1038/s41598-018-35253-2

**Published:** 2018-11-20

**Authors:** R. Burns, P. M. Wynn, P. Barker, N. McNamara, S. Oakley, N. Ostle, A. W. Stott, H. Tuffen, Zheng Zhou, F. S. Tweed, A. Chesler, M. Stuart

**Affiliations:** 1 Lancaster Environment Centre, University of Lancaster, Lancaster, LA1 4YQ UK; 2 Centre for Ecology and Hydrology, Lancaster, LA1 4AP UK; 3 Geography, Staffordshire University, College Road, Stoke-on-Trent, Staffordshire, ST4 2DE UK; 4 School of Earth and Climate, The University of Maine, 5790 Bryand Global Sciences Center, Orono, ME 04469 USA

## Abstract

The base of glaciers and ice sheets provide environments suitable for the production of methane. High pressure conditions beneath the impermeable ‘cap’ of overlying ice promote entrapment of methane reserves that can be released to the atmosphere during ice thinning and meltwater evacuation. However, contemporary glaciers and ice sheets are rarely accounted for as methane contributors through field measurements. Here, we present direct field-based evidence of methane production and release from beneath the Icelandic glacier Sólheimajökull, where geothermal activity creates sub-oxic conditions suited to methane production and preservation along the meltwater flow path. Methane production at the glacier bed (48 tonnes per day, or 39 mM CH_4_ m^−2^ day^−1^), and evasion to the atmosphere from the proglacial stream (41 tonnes per day, or 32 M CH_4_ m^−2^ day^−1^) indicates considerable production and release to the atmosphere during the summer melt season. Isotopic signatures (−60.2‰ to −7.6‰ for δ^13^Cch_4_ and −324.3‰ to +161.1‰ for Dch_4_), support a biogenic signature within waters emerging from the subglacial environment. Temperate glacial methane production and release may thus be a significant and hitherto unresolved contributor of a potent greenhouse gas to the atmosphere.

## Introduction

The subglacial environment provides conditions suitable for the production and storage of methane. The presence of liquid water beneath temperate and polythermal ice masses, sub-oxic conditions due to poor hydrological connectivity, and carbon within basal sediments allow the survival of microbiological communities with the potential to produce biogenic methane eg.^1–3^. The source of carbon essential for fuelling microbiologically-mediated reactions can be generated either *in-situ* at the bed of the glacier through chemolithoautotrophic production^4^, through utilising organic containing fossil soils^2,5–7^, or sourced from the surface environment^8^. Geogenic subglacial methane comprises abiogenic sources from subglacial volcanism, geothermal activity, and thermogenic sources through the thermal degradation of organic matter and subsequent migration of methane from hydrocarbon reserves to a stable sub-ice storage location e.g.^3,9^. Methane present at the bed of glaciers and ice sheets can then be retained *in situ* by high overburden pressures and low temperatures, which promote the entrapment of gases, or the storage of methane in hydrate reserves. Vast methane reservoirs potentially containing hundreds of petagrams of carbon could therefore accumulate and, if released as the ice melts, contribute positive feedback to rising atmospheric greenhouse gas concentration and global temperature^1–3^.

However, despite the presence of active microbial assemblages^5,8,10^ and favourable pressure–temperature relationships^1–3^ for methane production and storage in the subglacial realm, methane in glacial meltwaters has only been directly detected as aqueous methane in one study (albeit with limited sampling regime)^11^, or else inferred indirectly using δ^13^C of dissolved organic carbon^12^. For methane to be detected in meltwater outflow, sub-oxic conditions must occur not only at the glacier bed, but also throughout the subglacial drainage path. These conditions typically arise at the onset of subglacial discharge, during winter baseflow or as brief reversals in redox status of subglacial waters^11,13–18^. The inherently transient nature of these conditions thereby provides limited opportunity for direct field-based characterisation of methane production. Here, we examine an Icelandic glacier, Sólheimajökull, an outlet glacier of the Mýrdalsjökull icecap (Supplementary Fig. S1), where sub-oxia within the subglacial water column allows the transport of methane from beneath the ice mass and enables isotopic determination of methane formation mechanisms. The subglacial hydrological system of Sólheimajökull supports extensive sub-oxic conditions throughout the summer due to deep connectivity with the geothermal zone of the active, ice covered Katla volcanic system, where release of reduced gases consume oxygen in the meltwaters^19^. Sub-oxic summer discharge thereby preserves dissolved methane during transport from beneath the glacier. Here, we document the changing concentration and isotopic composition of methane contained within glacial waters across the 2013 to 2017 melt seasons in order to determine methane flux and mechanism of formation. Stable isotopic analysis of δ^13^Cch_4_ and Dch_4_ are used to provenance the methane between biogenic and geogenic sources, and incubation experiments are used to support field evidence for methane biogeochemical cycling. Whilst the specific environmental conditions at Sólheimajökull provide ideal opportunities to investigate mechanisms of methane formation and release dynamics, they also highlight the potential for methane production beneath contemporary glaciers worldwide, especially under a changing climate.

## Results

**Figure 1:**
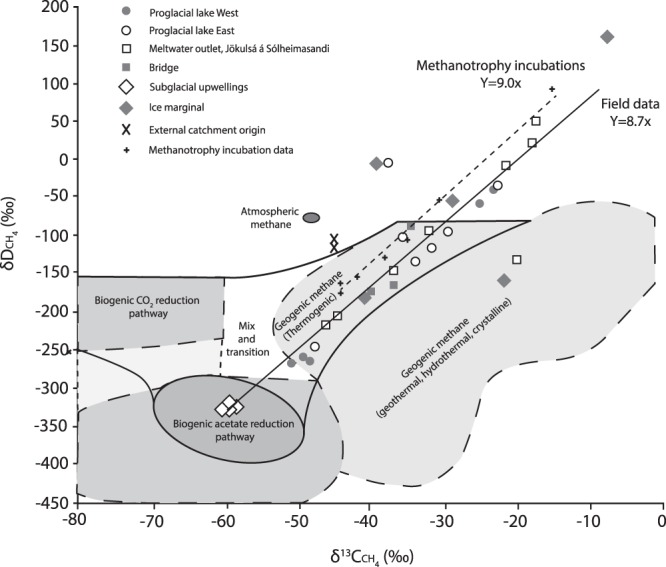
Carbon and hydrogen isotopic composition of methane in field data from Sólheimajökull, Iceland and in residual headspace gases during incubation of subglacial sediments under methanotrophic conditions. Bounded areas represent the typical range in methane isotopic composition of microbial and geogenic origin, modified from^26^. The similar relationship between δ^13^Cch_4_ and Dch_4_ in incubations and field data suggest the presence of methanotrophic activity within the field environment.

Concentrations of methane present in the meltwater streams of Sólheimajökull between 2013 to 2017 are provided in Table 1. Greatest methane concentrations coincide with the location of meltwaters upwelling under pressure from the subglacial environment. Streams originating from external catchments and those of supraglacial source contain minimal levels of aqueous methane, with limited contribution to the total methane flux. Methane concentrations also vary on a seasonal basis, with greatest concentrations apparent following upwelling of subglacial meltwaters in the late melt season (Table 1). Field chamber-based experiments demonstrate minimal methane production and consumption from the proglacial sediments (Supplementary Table S1). Isotope signatures of δ^13^Cch_4_ and Dch_4_ measured *in-situ* in meltwaters from the 2014 field season have values ranging between −60.2‰ to −7.6‰ for δ^13^Cch_4_ and −324.3‰ to +161.1‰ for Dch_4_ (Fig. 1). At the point of subglacial upwelling, methane appears to be of predominantly microbial origin (δ^13^Cch_4_ < −50‰) and laboratory incubation of associated subglacial sediments demonstrate a strong potential for methanogenesis (methane production rates of 1.15 × 10^7^ fmol CH_4_ g^−1^ h^−1^ at incubation temperatures of 15 °C; see Supplementary Fig. S2). Potential for methanotrophy within the subglacial sediments is also demonstrated through incubation of sediments under oxidising conditions (methane oxidation rates of 9.6 × 10^9^ fmol CH_4_ g^−1^ h^−1^, at 15 °C, see Supplementary Fig. S2).

**Table 1:** Methane concentrations, isotopic values and metadata from aqueous samples collected in the Sólheimajökull forefield between 2013 to 2017. Values presented are mean compositions collected from different field locations pre and post emergence of subglacial waters. The range of values is given in parentheses with sample number presented as (*n*=).

Sampling location	Pre-upwelling (up until DOY 128)			Early post upwelling (immediately post DOY 128)			Late post upwelling (From DOY 185)		
	CH_4_ (ppm)	δ^13^Cch_4_	δDch_4_	CH_4_ (ppm)	δ^13^Cch_4_	δDch_4_	CH_4_ (ppm)	δ^13^Cch_4_	δDch_4_
2013									
Supraglacial							0.14 (n = 2) (0.12 to 0.15)	n.d	n.d
Meltwater outlet, Jökulsá á Sólheimasandi							15.2 (n = 8) (5.95 to 20.78)	−56.4 (n = 4) (−57.12 to −56.03)	n.d
Proglacial lake East							8.17 (n = 9) (0.80 to 18.14)	−53.8 (n = 9) (−57.13 to −47.58)	n.d
Proglacial lake West							12.2 (n = 2) (11.59 to 12.75)	−56.6 (n = 2) (−56.76 to −56.45)	n.d
Catchment outlet (Bridge)							4.2 (n = 2) (3.88 to 4.62)	−49.5 (n = 2) (−51.58 to −47.35)	n.d
Subglacial upwelling							n.d	n.d	n.d
Streams of external catchment origin							0.17 (n = 4) (0.12 to 0.27)	n.d	n.d
2014									
Supraglacial	0.33 (n = 2) (0.27 to 0.40)	n.d	n.d	0.27 (n = 1)	n.d	n.d			
Meltwater outlet, Jökulsá á Sólheimasandi	0.65 (n = 7) (0.46 to 0.78)	−22.5 (n = 7) (−27.9 to −17.93)	+22.9 (n = 1)	1.23 (n = 8) (0.47 to 1.95)	−39.6 (n = 6) (−46.38 to −32.27)	−166.9 (n = 4) (−218.3 to −95.9)	7.51 (n = 2) (3.77 to 6.57)	−55.98 (n = 2) (−55.28 to −56.68)	n.d
Proglacial lake East	1.05 (n = 17) (0.36 to 3.21)	−27.8 (n = 8) (−36.98 to −15.91)	−96 (n = 4) (−134.2 to −7.2)	1.4 (n = 6) (0.28 to 3.82)	−42.9 (n = 6) (−47.84 to −35.82)	−174.1 (n = 2) (−246.4 to −101.7)			
Proglacial lake West	1.91 (n = 3) (1.46 to 2.37)	−25.3 (n = 1)	−59.2 (n = 1)	2.86 (n = 4) (1.13 to 4.99)	−41.5 (n = 3) (−51.61 to −23.17)	−189.1 (n = 3) (−267.2 to −39.8)			
Catchment outlet (Bridge)	0.32 (n = 3) (0.32 to 0.33)	−34.2 (n = 1)	n.d	1.74 (n = 3) (0.36 to 3.11)	−37.3 (n = 3) (−40.4 to −34.57)	−141.2 (n = 3) (−174.1 to −86.6)			
Subglacial upwelling	n.d	n.d	n.d	17.57 (n = 6) (11.71 to 21.73)	−59.6 (n = 6) (−60.22 to −58.56)	−323.7 (n = 4) (−324.3 to −322.6)			
Streams of external catchment origin	0.26 (n = 2) (0.26 to 0.27)	n.d	n.d	0.28 (n = 4) (0.26 to 0.30)	−44.9 (n = 4) (−46.25 to −42.85)	−108.8 (n = 2) (−112.6 to −104.9)			
2017									
Supraglacial									
Meltwater outlet, Jökulsá á Sólheimasandi							10.87 (n = 3) (7.66 to 12.75)	n.d	n.d
Proglacial lake East							4.12 (n = 7) (0.14 to 7.46)	n.d	n.d
Proglacial lake West									
Catchment outlet (Bridge)							0.25 (n = 1)	n.d	n.d
Subglacial upwelling									
Streams of external catchment origin

## Discussion

The appearance of elevated aqueous methane concentrations that are commensurate with the location and onset of subglacial drainage, suggests the environment of methane production must be beneath the glacier. Fluctuating discharge and changing subglacial methane concentrations on a seasonal basis preclude straightforward calculation of an annual methane flux from beneath the glacier. However, a typical summer season discharge of 50 m^3^ s^−1^ from the meltwater outlet stream Jökulsá á Sólheimasandi^20^, and the corresponding average aqueous methane concentration of 11.2 mg l^−1^ (Table 1) can be used to estimate a flux of 48 Tonnes per day of methane transported away from the ice margin. This high flux occurs as meltwater exits the ice-marginal proglacial lake after the onset of discharge from the subglacial drainage system. When calculated as a day-rate per m^2^ ice-covered area (maximum 78 km^2^ glaciated catchment area cf.^21^) this equates to a subglacial production capacity of 39 mM CH_4_ m^−2^ day^−1^. Using an upstream – downstream mass balance along the 4 km length (20 m width) of proglacial meltwater channel (taken as the difference in methane concentration between the meltwater outlet sampling site and the catchment outlet at the N1 road bridge, supplementary Fig. S1), evasion to the atmosphere was calculated as 86%. This equated to an evasive flux of 41 tonnes of methane to the atmosphere per day (32 M CH_4_ m^−2^ day^−1^ as an area-weighted flux from stream to atmosphere). This mass balance approach to calculating an evasive methane flux along the stream assumes minimal dilution, and no in-stream methanotrophy. Both are valid assumptions given the minimal input of additional meltwater between upstream and downstream sampling points, the minimal production/consumption of methane in the proglacial sediments (Table S1), and the limited change in isotopic composition of aqueous methane (Table 1). The onset of upwelling subglacial water varies on an annual basis at all glaciers, dependent on antecedent conditions. Prior to the upwelling of subglacial meltwaters during the 2014 sampling season (day of year 128), a more conservative flux of methane transported away from the ice margin is estimated as 0.6 tonnes per day (equivalent to 0.5 mM CH_4_ m^−2^ day^−1^, based on an average winter discharge of 10 m^3^ s^−1^ and mean methane concentration of 0.65 mg l^−1^). Evasion to the atmosphere was calculated as 54% along the 4 km stream reach, equating to 0.25 M CH_4_ m^−2^ day^−1^. Methane evasion from the Sólheimajökull sub-aerial stream network greatly exceeds mean flux values between river to atmosphere reported in the literature (4.23+\−8.41 mM CH_4_ m^−2^ day^−1^)^22^ indicating the potential significance of the subglacial methane source, if similar processes are also occurring at other glaciers.

The origin of the methane can be inferred through stable isotopic analysis of δ^13^Cch_4_ and Dch_4_. Isotopic fractionation during biogenic methanogenesis typically leads to δ^13^C values between −50 to −110‰, and δD values between −170 to −531‰^23^. Geogenic methane produced at high geothermal temperatures undergoes exchange with the surrounding water and mantle carbon, producing deuterium and carbon contents enriched in ^2^H and ^13^C respectively^24^. Signatures of mixed geogenic/microbial origin should therefore lie on an end member mixing trajectory as depicted in Fig. 1, with microbially-sourced methane clearly emanating from the point of subglacial upwelling. However, possible alteration to methane signatures by methanotrophic activity (methane oxidation) will enrich the remaining pool of methane reactants in ^13^C and deuterium. As the most enriched values exceed the geogenic range, the observed isotopic signatures cannot be explained by a mixture of biogenic and geogenic methane (Fig. 1). Extensive potential for methanotrophic activity, as evidenced through the incubation of sediments under oxidizing conditions (see Supplementary Fig. S2), likely explains the isotopic fractionation trajectory away from the microbial end member signature. Fractionation between the starting methane isotopic composition (CH_4(i)_) and composition of residual methane (CH_4(t)_) is quantified following^25^ as α = 1.019 for ^13^C/^12^C, and for fractionation of D/H as α = 1.197. These incubation determined values of C and H enrichment during methanotrophy are encompassed within the published range of experimental values^23^, and result in relative changes to isotopic signatures during reaction progress that lie on a similar gradient to field data from this study (Fig. 1 and methods). This isotope signature confirms that methane emanating from the subglacial environment of Sólheimajökull is predominantly regulated by microbial activity.

**Figure 2:**
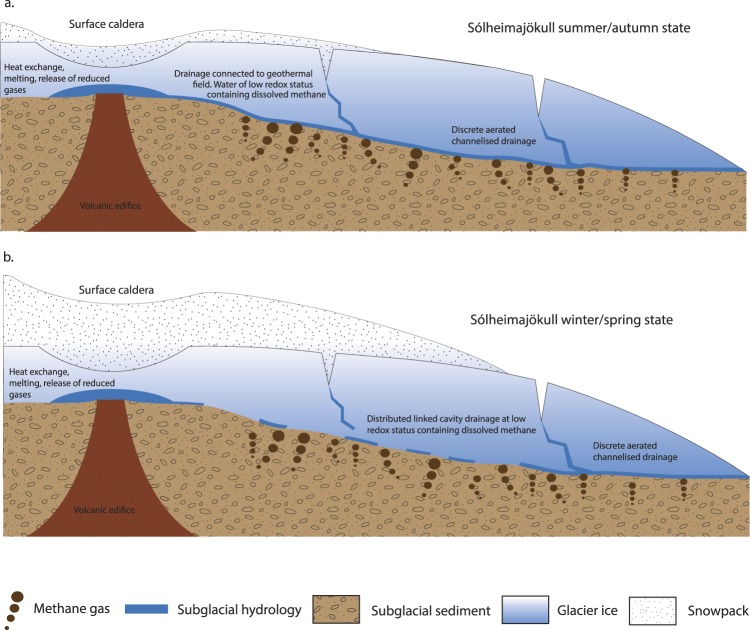
Schematic model of hydrological evolution at Sólheimajökull, Iceland. The headward expansion of the conduit drainage system intersects with the geothermal area, where release of reduced gases determines the sub-oxic meltwater status essential for preserving aqueous methane until the point of emergence from beneath the glacier. **(a)** Summer season snowpack ablation delivers meltwater to expand the conduit drainage system headwards into the Katla geothermal field. This results in a drainage system well-connected to deep within the geothermal field, delivering water of reducing status. Methane generated within the basal sediments through microbial methanogenesis is preserved during export. **(b)** Winter season limited surface ablation restricts the conduit drainage system to the lower reaches of the glacier. This results in a drainage system operating close to atmospheric conditions within the vicinity of the glacier snout and poorly connected to deeper beneath the ice mass. Much of the methane generated within the surrounding sediments is oxidised proximal to the channelized drainage system before being exported from beneath the glacier.

The important role played by microbial activity in determining this remarkably high methane flux from beneath Sólheimajökull is surprising given the extensive geothermal activity beneath the Mýrdalsjökull icecap^26^. However, based on isotopic evidence, subglacial geothermal activity appears not to contribute to the methane flux. Instead, we consider the subglacial geothermal activity to be instrumental only in driving the summer subglacial discharge to low redox status, allowing preservation and transport of microbially-generated, dissolved methane to the point of upwelling without oxidation to CO_2_. Most temperate glacial drainage systems which do not overlie volcanic and/or geothermal systems are characterised by a slow flow winter component in which subglacial water is confined to linked cavities, basal film flow and/or water saturated till, dependent upon the state of the glacier bed (hard- or soft-based). Under these conditions of distributed drainage (the ‘closed’ system), connectivity to the atmosphere is poor and dissolved gases can be depleted to produce meltwaters of low redox status. During the summer season, a discrete well-connected subglacial drainage system, characterised by well-defined conduits, expands up-glacier dependent upon the flux of surface run-off to the glacier bed, and typically follows the supraglacial snowline. Within this ‘open’ configuration, oxygen saturated meltwaters can drain rapidly from the surface of the glacier and through the subglacial system^27,28^. At polar glaciers of a polythermal nature, the drainage system displays similar characteristics, albeit with the winter slow flow component of the drainage system remaining sealed beneath the glacier until basal water pressures force a pressurised outflow, either shortly after the onset of the summer season^29^, or intermittently throughout the winter to produce characteristic proglacial icings e.g.^30^. However, at Sólheimajökull, the presence of the Katla geothermal area beneath the head of the glacier imparts profoundly different characteristics to meltwater discharge (Fig. 2). During the summer season (Fig. 2a), headward expansion of the conduit drainage system proceeds in the conventional fashion based on an enhanced flux of meltwater between glacier surface and bed. When the conduit drainage system connects with the zone of geothermal activity, release of reduced gases into the drainage system produces the characteristic volatile-rich, oxygen-depleted chemical composition of the discharge, as evidenced by the hydrogen sulphide content and sulphur isotopic composition of the meltwaters^19^. The summer season sub-oxic meltwater arguably inhibits methanotrophic activity beneath the glacier, allowing the preservation of dissolved biogenic methane until the point of upwelling and contact with the atmosphere. The transported methane comprises methane formed during the winter ‘closed’ system phase (zero-flux scenario^3^), together with methane produced during the summer season. During the winter season (Fig. 2b), the conduit drainage system is restricted to the lower elevations of the glacier, where year-round ablation maintains a conduit configuration connected to the atmosphere and isolated from the Katla geothermal zone. Under this configuration, methane production is limited and methanotrophic activity minimises the methane flux.

The geothermally-influenced nature of the Sólheimajökull system is unusual in its ability to present a low redox status window which inhibits methane oxidation and preserves aqueous methane until the point of release from beneath the glacier. The dominance of biogenic methane production beneath an Icelandic temperate ice mass nonetheless raises the distinct possibility that methane generation could be proceeding undetected in other subglacial environments where the cocktail of temperate ice, low oxygen concentration, organic carbon and methanogenic communities coincide to promote methanogenesis. Increasing evidence for zones of strong geothermal activity beneath the West Antarctic Ice Sheet suggests that subglacial microbial communities with methanogenic potential may be more significant and extensive than previously anticipated^31,32^. However, cold ice barriers and the length of meltwater pathways to ice termini means methane is typically trapped beneath ice masses, or oxidised during subglacial transit away from its zone of production. This prevents confident extrapolation of the subglacial methane production at Sólheimajökull to other regions, or to a global scale. Thus, the relative contribution of subglacial methane to global atmospheric fluxes critically depends on the extent of sub-oxic ‘windows’ at temperate and polythermal basal ice systems. We suggest that, in order to identify subglacial methane fluxes from temperate and polythermal glacial systems, and better constrain any associated climatic impact, the quest for quantifying methane release dynamics should focus on these sub-oxic windows of meltwater discharge. This may include studying other ice masses with elevated geothermal heat fluxes; characterising baseflow seepage and initial fractions of summer subglacial discharge at both temperate and polythermal glaciers; and analysing gases trapped within proglacial icings. Under a changing climate with accelerated ice thinning^33^, enhanced overburden pressure release on subglacial volcanic and geothermal systems is likely to drive an increase in eruptive activity^34^. Anomalous heat fluxes are known to both precede and follow volcanic activity, likely due to pressure-induced boiling in geothermal systems^35^. Release of reduced gases during this enhanced geothermal activity would determine the prevalence of sub-oxic windows (and methane content) of meltwater discharge. Greater headward expansion of drainage systems towards geothermal areas currently isolated beneath ice mass overburden would also ensure transport of meltwater and associated methane content to a position of sub-aerial degassing into the atmosphere. Pressure driven sub-oxia would likely become more prevalent with ice thinning until overwhelmed by the flux of oxygenated surface melt reaching the glacier bed or until ice disappearance. In this manner, this poorly quantified flux of sub-ice cap methane is likely an indirect, albeit self-reinforcing consequence of climatic change.

## Methods

### Sample collection

Field samples were collected over restricted periods within the melt seasons of 2013 to 2017. Precise collection intervals are depicted in Table 1, with the 2014 collection period noted to cover both pre- and post-emergence of subglacial upwelling meltwaters. As far as the evolving nature of the proglacial system allowed, sample collection sites remained at consistent locations throughout both summer seasons. Sampling locations for aqueous methane comprised supraglacial streams, subglacial upwellings located at the frontal ice margin, and proglacial waters sampled at sites flanking the eastern and western edges of the ice marginal proglacial lake, and as mixed meltwaters in the outlet stream, Jökulsá á Sólheimasandi. Streams of external catchment origin were sampled as control sites to verify methane as specific to the Sólheimajökull catchment. Repeat samples were collected at each location throughout the period of monitoring.

Samples for the determination of aqueous methane concentration were collected as a known volume of unfiltered water and stored within an airtight chamber with headspace at atmospheric pressure. Samples were left for 24 hours to undergo headspace equilibration and gases were then extracted through a sampling port and injected into evacuated exetainers (Labco Ltd, UK) for later analysis of methane concentration and isotopic determination. Exetainers were over-pressurised to prevent ingress of atmospheric air and stored at ambient temperature to prevent vessel contraction and leakage. Headspace gas extraction at time t = 0 was used to determine background concentration prior to sample equilibration.

Proglacial sediments were monitored for the production and consumption of methane (methanogenesis and methanotrophy respectively) using static chambers (15 cm diameter × 10 cm height). Chambers were inserted into the sediments in triplicate at each site, and covered in aluminium foil to minimise temperature changes during the sampling period. Headspace gases were removed at set time intervals over a 45 minute incubation period to monitor the rate of methane production/consumption. Headspace gases were injected into evacuated exetainers which were over-pressurised to prevent the ingress of atmospheric air and stored at ambient temperature prior to further analysis for methane concentration. Flux values were calculated as µM CH_4_ m^−2^ day^−1^ following^36^.

### Incubation procedure

Sediments extruded onto the glacier surface via thrust planes or melt out of fracture fill deposits c.f.^37^ were deemed the closest analogue to typical subglacial sedimentary deposits from the Sólheimajökull catchment. Sediments were incubated to determine the potential for methane production (methanogenesis) and consumption (methanotrophy) using standard procedures^38^. For each incubation type 10 grams of fresh weight sediment was added to a 100 ml sterilised Wheaton bottle and slurried with 20 ml deionised water. For anaerobic methane production incubations, the headspace was flushed with nitrogen gas to eliminate oxygen. For aerobic incubations the headspace was flushed with synthetic air, following which the methane concentration was adjusted to 150 ppm methane to assay for methanotrophy. Each set of incubations operated alongside control chambers supporting identical headspace conditions, but without the inclusion of sediment. All incubations were undertaken at a set temperature of 15 °C, reflecting optimal conditions for methane production and consumption, as established through preliminary testing. For methane production and oxidation potentials, triplicate samples were incubated for 49 and 7 days, respectively with regular sampling intervals during the periods of incubation (Supplementary Fig. S2). At the time of sampling 1 ml was withdrawn from the headspace and directly injected into the GC (see below for details of analysis). Rates of methanotrophy and methanogenesis were calculated based on a production potential per day, per gram (dry weight) of sediment. Samples for δ^13^C and δD analysis were withdrawn from the incubation chamber headspace and injected into pre-evacuated 3 ml exetainers (see below for details of isotopic analysis). Fractionation between starting methane isotopic composition (CH_4(i)_) and composition of residual methane (CH_4(t)_) in the closed headspace is calculated following^25^:

1 $$\alpha ={[\frac{\mathrm{ln}(\frac{\delta XC{H}_{4(t)}+1000}{\delta XC{H}_{4(i)}+1000})}{\mathrm{ln}f}+1]}^{-1}$$

Where *f* is the fraction of methane remaining and δX is the isotopic composition of methane.

### Laboratory Chemical analysis

Methane concentrations were analysed using a PerkinElmer Autosystem XL Gas Chromatograph (GC) (PerkinElmer, Waltham, MA, USA) fitted with a Flame Ionisation Detector (FID) operating at 300 °C. The GC was fitted with a stainless steel Porapak Q 50–80 mesh column (length 2 m, outer diameter 3.17 mm) maintained at 60 °C. Three calibration gas standards (1, 10, 500 ppm CH_4_) (Air Products, Waltham on Thames, UK) were analysed in every analytical sequence to encompass the expected sample concentrations. Standards were repeated at regular intervals^39^ to check for drift and ensure accuracy to within 95% of the true value. Sample triplicates had a precision (1S.D) representing <0.2% of the average value. The concentration of methane in water (C_aq_) is related to the concentration of gas measured in the headspace (C_g_) via the dimensionless Henry’s Law solubility Constant (H^CC^) at a temperature of 273 K (0 °C)^40^.

^13^C/^12^C and D/H ratios of headspace methane gas were determined by online combustion/pyrolysis respectively, followed by analysis using continuous flow isotope ratio mass spectrometry. For determination of δ^13^Cch_4_, headspace gases were injected manually into an Isoprime Trace gas analyser coupled to an Isoprime continuous flow isotope ratio mass spectrometer (Elementar UK, Stockport) at the NERC Life Sciences Mass Spectrometer Facility, CEH Lancaster, UK. Manual injection volumes were dependent upon methane concentration and did not exceed 10 ml. Samples were initially passed through a Magnesium perchlorate/Carbosorb scrubber trap at 20 ml/min to eliminate water and CO_2_. Methane is oxidised in a combustion furnace using a braided platinum/copper/nichrome furnace wire inside a ceramic furnace tube of 200 mm × 0.4 mm i.d. heated in a furnace at 960 °C^41^. A preparation flow rate of 10 psi was required to give a flow rate of 20 ml/min through the furnace at full operating temperature. For δDch_4_, gas samples were purged from vials using a dual core needle and Helium carrier gas into a ThermoScientific precon concentration unit interfaced to a ThermoScientific Delta V plus isotope ratio mass spectrometer at UC Davies, University of California, USA. Cryogenic trapping and GC separation followed by pyrolysis at 1450 °C yielded H_2_ for determination of D/H ratios of methane gas^42^. δ^13^C values were corrected to VPDB using working CH_4_ standards cross calibrated with a CO_2_ reference gas, calibrated to NIST REF-Heavy Palaeomarine Origin (CO_2_) (RM 8562) and NIST REF-Biogenic Modern Biomass Origin (CO_2_) (RM 8564). The reproducibility of δ^13^Cch_4_ was better than ±0.2‰. δD was corrected to VSMOW using reference gasses calibrated to international standards NIST 8559, 8560, and 8561. Within-run standard replication of both samples and standards (1SD) was better than 2.6‰ for Hydrogen.

## Electronic supplementary material

Supplementary information

## Data Availability

The underlying data pertaining to the figures and tables within this manuscript is available from 10.17635/lancaster/researchdata/246.
