# Supplementary material for: Direct isotopic evidence of biogenic methane production and efflux from beneath a temperate glacier
Source: Sci Rep. 2018 Nov 20;8:17118. doi: 10.1038/s41598-018-35253-2 (PMC6244297; doi:10.1038/s41598-018-35253-2)
Supplement: Supplementary file 1 — Supplementary information [file 41598_2018_35253_MOESM1_ESM.docx]

**Supplementary Information**

**Direct isotopic evidence of biogenic methane production and efflux from beneath a temperate glacier**

Burns, R^a^; Wynn PM^a*^; Barker, P^a^; McNamara, N^b^; Oakley, S^b^; Ostle, N^a^; Stott, AW^b^; Tuffen, H^a^; Zheng Zhou^a^; Tweed, FS^c^; Chesler, A^a,d^; Stuart, M^a^

*^a^Lancaster Environment Centre, University of Lancaster, Lancaster,*

*LA1 4YQ. UK.*

*^b^Centre for Ecology and Hydrology, Lancaster, LA1 4AP. UK*

*^c^ Geography, Staffordshire University, College Road, Stoke-on-Trent, Staffordshire, ST4 2DE. UK*

*^d^School of Earth and Climate, The University of Maine, 5790 Bryand Global Sciences Center,
Orono, ME 04469. USA*

* Corresponding author. Tel. +44(0)1524 510235. E-mail address: [p.wynn@lancaster.ac.uk](mailto:p.wynn@lancaster.ac.uk)


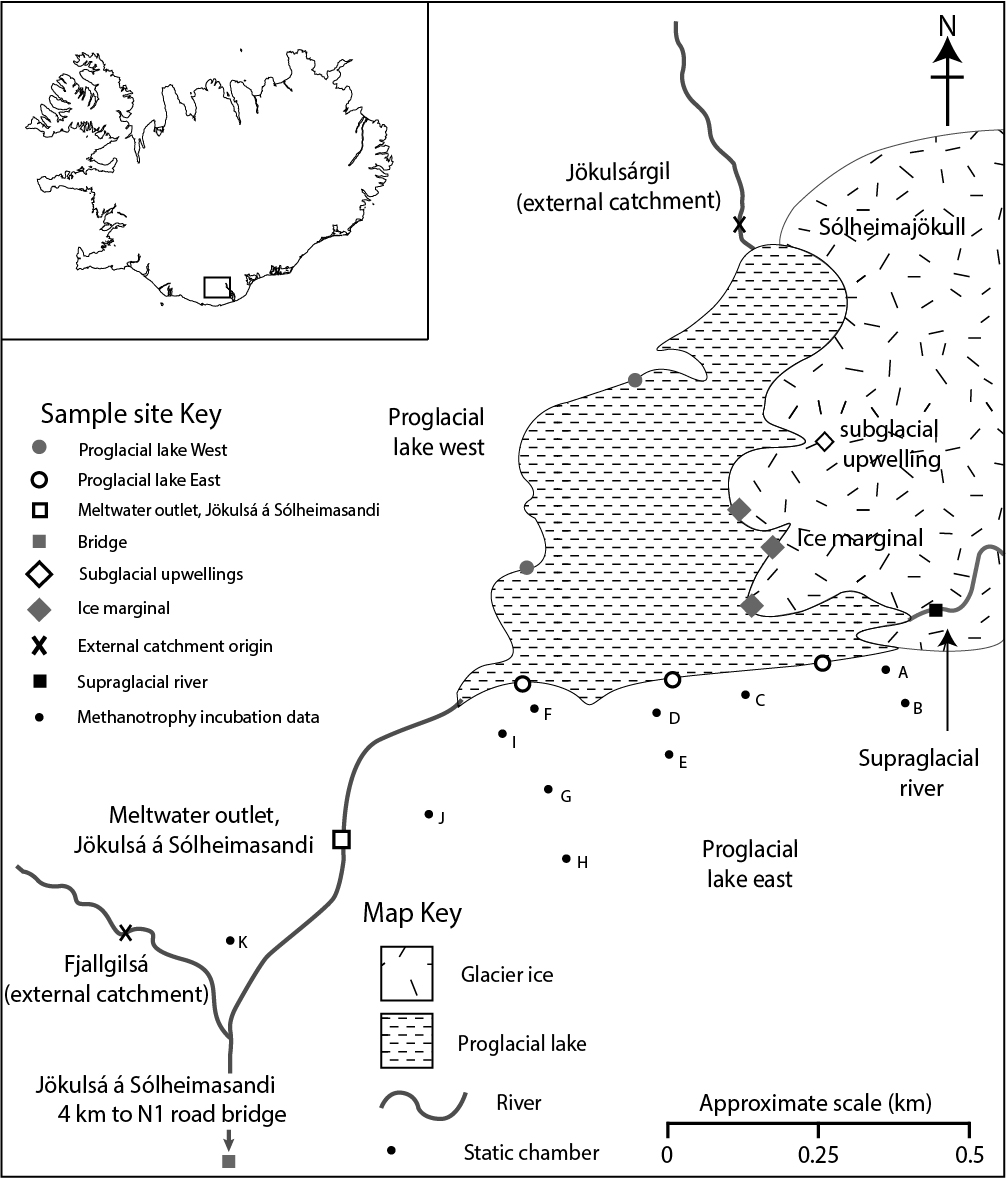


**Supplementary Figure S1:** Location map of Sólheimajökull, Iceland, site characteristics and sampling locations during 2013-2017. Sólheimajökull is an Icelandic maritime glacier located on the south coast of Iceland, draining the Mýrdalsjökull ice cap. It is 8 km long, temperate, non-surging, and has a maximum ice-covered catchment area of 78 km^2^ that extends from the ice-marginal proglacial lake towards the southwestern rim of the Katla subglacial caldera.

**Supplementary Figure S2:** Incubation of subglacial sediments to determine rates and characteristics of both methane production (methanogenesis) and methane consumption (methanotrophy). **S2a:** Methane production from incubated sediments over a 49-day incubation period. Extrapolated methane production rate of 1.1 x 10^7^ fmol per g dry weight sediment per hour. This production rate compares to other published values ranging between 126 fmol g^-1^ hr^-1^ (incubations undertaken at 15°C^[10]^); to 10^5^ fmol g^-1^ hr^-1^ (incubations undertaken at 10°C^[9]^) in Canadian and Antarctic sediments respectively. **S2b:** Methanotrophic activity over a 7-day incubation period. Extrapolated methane consumption rate of 9.6 x 10^9^ fmol per g dry weight of sediment per hour. **S2c:** The isotopic fractionation trajectory of δ^13^CCH_4_ and DCH_4_ during incubation of sediments to stimulate methanotrophic activity. **S2d:** Changes in the carbon and hydrogen isotopic value of residual methane during incubation and methanotrophy of subglacial sediments. Fractionation between the starting methane isotopic composition (CH_4(i)_) and composition of residual methane (CH_4(t)_) is quantified as α=1.019 for fractionation of ^13^C/^12^C, and for fractionation of D/H as α=1.197 (see methods in main manuscript for calculation). The calculated gradient is compared to field data (this study) in Figure 1.

**Table S1:** Methane flux data from static chambers located within the proglacial sedimentary deposits of Sólheimajökull. Data are presented as µM CH_4_ m^-2^ day^-1^ with the range of values given in parentheses and number of replicates presented as *n =*. The location of each experimental chamber is given in Supplementary Figure S1.

| **Static chamber** | **Day of year 2013**  **(µM CH_4_ m^-2^ day^-1^)** | | | | | | | **Day of year 2014**  **(µM CH_4_ m^-2^ day^-1^)** |
| --- | --- | --- | --- | --- | --- | --- | --- | --- |
|  |  | | | | | | |  |
|  | **187** | **190** | **192** | **195** | **196** | **198** | **203** | **136** |
| **A** |  | -0.0054  (-0.0264 to 0.0059)  n=3 |  | 0.0392  (-0.0327 to 0.1256  n=3 |  |  |  | -0.0301  (-0.0442 to -0.0143  n=3 |
| **B** |  |  |  |  | 0.000  (-0.0057 to 0.0029  n=3 |  |  |  |
| **C** |  | 0.0055  (-0.0038 to 0.0008)  n=3 |  | 0.0019  (-0.0155 to -0.0082)  n=3 |  |  |  | 0.0033  (-0.0047 to 0.0099)  n=3 |
| **D** |  |  |  |  | -0.0058  (-0.0082 to -0.0041)  n=3 |  |  |  |
| **E** |  |  |  |  |  | -0.0047  (-0.0091 to 0.0070)  n=3 |  |  |
| **F** | -0.0258  (-0.0552 to 0.0033)  n=3 | 0.0355  (0.0341 to 0.0828)  n=3 | -0.0092  (-0.0191 to 0.0072)  n=3 | -0.0041  (-0.0114 to 0.0000)  n=3 |  |  |  | -0.0781  n=1 |
| **G** |  |  | 0.0160  (-0.0008 to 0.0322)  n=3 | 0.0023  (-0.0007 to 0.0060)  n=3 |  |  |  |  |
| **H** |  |  | 0.0009  (-0.0086 to 0.0141)  n=3 | -0.0002  (-0.0070 to 0.0039)  n=3 |  |  |  |  |
| **I** |  |  | -0.0038  (-0.0139 to 0.0029)  n=3 |  |  |  |  |  |
| **J** |  |  |  |  |  | 0.0069  (-0.0124 to 0.04234)  n=3 |  |  |
| **K** |  |  |  |  |  |  | 0.0053  (0.0016 to 0.0089)  n=3 |  |
